# Individual and structural correlates of willingness for intravenous buprenorphine treatment among people who inject sublingual buprenorphine in France

**DOI:** 10.1186/s12954-021-00460-0

**Published:** 2021-01-19

**Authors:** Salim Mezaache, Patrizia Carrieri, Laélia Briand-Madrid, Virginie Laporte, Alain Morel, Daniela Rojas Castro, Perrine Roux

**Affiliations:** 1 INSERM, IRD, SESSTIM, Sciences Économiques & Sociales de La Santé & Traitement de L’information Médicale, Aix-Marseille Univ, Marseille, France; 2 ORS PACA, Observatoire Régional de La Santé Provence-Alpes-Côte D’Azur, Marseille, France; 3 Aides, Pantin, France; 4 Association Oppelia, Paris, France; 5 Laboratoire de Recherche Communautaire Coalition PLUS, Pantin, France

**Keywords:** Opioid-related disorders, Opiate substitution treatment, Intravenous substance abuse, Harm reduction

## Abstract

**Background:**

Some people do not benefit from oral administration of opioid agonist treatment, and an intravenous (IV) formulation may be more suitable. Our objective was to evaluate the willingness of people who regularly inject sublingual buprenorphine to receive IV buprenorphine as a prescribed treatment, and to examine related correlates.

**Methods:**

We performed a secondary analysis of data from the cross-sectional study PrebupIV, conducted in France in 2015 among 557 people who inject opioids. The study comprised questionnaires completed either face to face or online and community-based workshops. We only included participants who reported buprenorphine as their main injected drug (*n* = 209). Willingness to receive IV buprenorphine treatment was measured on a scale from 0 to 10. Ordinal logistic regression identified correlates of willingness. Artworks and testimonies from participants in the workshops were also used to illustrate correlates of willingness.

**Results:**

Among the 209 participants, the mean score (SD) for willingness to receive IV buprenorphine was 8.0 (2.8). Multivariate analysis showed that participants who reported using non-prescribed buprenorphine (AOR = 4.82, *p* = 0.019), a higher daily dosage of buprenorphine (AOR (for 1 mg) = 1.05, *p* = 0.043), and a higher number of complications due to injection (AOR = 2.28, *p* = 0.037), were more willing to receive IV buprenorphine treatment.

**Conclusions:**

Willingness to initiate IV buprenorphine treatment was high among people who regularly inject sublingual buprenorphine. A prescribed IV formulation could attract and retain more people into care and reduce harms associated with the injection of buprenorphine tablets.

## Background

In many high-resource countries, people with opioid use disorder (OUD) have access to opioid agonist treatment (OAT) through various access modalities. Oral methadone and sublingual buprenorphine are considered gold standards for OAT as both are effective in reducing illicit opioid use and increasing treatment retention [1, 2]. Accessibility and retention in OAT treatment have also been associated with reduced morbidity and mortality (in particular regarding overdoses and viral infections) in people who use drugs, as well as improved social outcomes [3–5]. In France, naloxone-free sublingual buprenorphine (i.e., Subutex® and generics) has been widely available in primary care settings since 1996 thanks to its good safety profile [6]. In contrast, methadone initiation is still restricted to specialized addiction centers [7]. Easy and widespread access to prescribed sublingual buprenorphine has been highly effective in reducing opioid-related overdoses and HIV prevalence in France [8]. However, it is also associated with inappropriate use of buprenorphine, such as the use of higher doses, intravenous use, recreational use and illegal acquisition [9]. These behaviors, reported in many countries, may lead to various adverse outcomes including treatment failure, overdose, infectious diseases (e.g., HIV, HCV, abscesses) and other medical complications (e.g., puffy hands syndrome, thrombosis) [10–12]. The main people-reported motivation for inappropriate use in several reports, is self-treatment for withdrawal or addiction [13, 14]. This suggests that the treatment needs of the drug-injecting population are not being met. To date, numerous strategies using different buprenorphine formulations have been developed in an attempt to reduce inappropriate use. The first was to make the drug ineffective when used parenterally but still effective when used sublingually, by adding a µ-opioid receptor antagonist (i.e., naloxone) [15]. This strategy has shown inconsistent results especially in contexts where buprenorphine injection is already strongly entrenched [16]. More recently, greater focus has been placed on prolonged-release buprenorphine formulations such as transdermal patches, subdermal implants and subcutaneous depots, which remain active from a few days to 6 months [17]. These formulations have the potential to overcome weaknesses of sublingual buprenorphine—including poor bioavailability, patient forgetfulness and therapy supervision—and to prevent use of other opioids during OAT. They may be more suitable for some individuals than others, in particular people who are more socially integrated and are not using full µ opioid agonists [18]. Nevertheless, they do not completely meet the needs of people unable to stop daily intravenous injecting, including those dependent on the actual act of self-injecting, and those who continue to use opioids occasionally while on OAT. Prescribed intravenous OAT with diacetylmorphine (i.e., pharmaceutical heroin), and more recently hydromorphone, have been shown to satisfy the needs of this group of people who don’t benefit from conventional treatments [19, 20]. In addition, intravenous OAT are valuable harm reduction tools, preventing people from using uncontrolled and illegal street drugs, reducing criminal justice involvement or incarceration and improving social functioning [21]. However, these highly structured treatments are only currently available in a limited number of countries, excluding France, and access is conditional on daily attendance in specialized clinics where injection is supervised by medical practitioners. Treatment with IV buprenorphine may not only be a valid alternative to these two treatments, but also the first step in a treatment strategy pathway whereby users could choose to switch to non-injectable treatment if they wished. Moreover, dispensing IV buprenorphine could include the possibility of take-home doses for stabilized patients, thanks to its safety profile. Finally, as buprenorphine is less controlled than diacetylmorphine-based treatments, it potentially faces fewer political and regulatory barriers [19]. This is the reason why public health researchers, clinical experts and the PWID community, came together in 2015 to set up a project to evaluate the feasibility and efficacy of IV buprenorphine as a prescribed treatment in France. The first step of this project was to implement a community-based research study, entitled PrebupIV, to characterize people who inject opioids, and to evaluate their willingness to receive this potential treatment [22]. The community-based aspect of PrebupIV focused on involving people who inject opioids in the development of IV buprenorphine as a treatment, through their participation in the research process and dissemination of the results. Primary analyses of the study showed that buprenorphine injectors were more willing to receive IV buprenorphine than other opioid injectors [22]. Given this result and the fact that buprenorphine injectors will be the primary target group of this treatment in future clinical evaluations, we performed a sub-study among regular buprenorphine injectors. To identify which factors may lead to a greater acceptability of a potential new treatment, we sought to identify individual and structural factors correlated with willingness to receive IV buprenorphine for OUD.

## Methods

### Study design

PrebupIV is a community-based cross-sectional survey implemented in France in 2015 [22]. Data were collected between May and August 2015, either through quantitative questionnaires administered face to face in harm reduction programs, addiction centers, and primary care settings, or through a dedicated online questionnaire on the website Psychoactif.org. Details on how participants were solicited can be found elsewhere [22]. Inclusion criteria were having injected opioids at least once during the previous week, being aged 18 years and over, and being able to read and understand French. People who inject opioids were involved throughout the research process, from reviewing research questions and questionnaires at the beginning, to participating in the interpretation and dissemination of the results.

### Study population

Among the 557 participants in PrebupIV, we first excluded those who had no lifetime history of OAT (*n* = 32) and those who injected opioids fewer than 4 times a week (*n* = 154). This cutoff was in line with different clinical studies on injectable diacetylmorphine where the opioid use eligibility criterion ranged from opioid use in more than half the days during the previous three months to daily opioid use. We secondarily selected only those who reported that buprenorphine, prescribed or not, was the opioid they injected most (*n* = 216). Finally, we excluded 7 participants due to missing data on primary outcomes, yielding a total study sample of 209 participants.

### Measurements

Data were collected using a purpose-built 31-item questionnaire divided into 3 sections: 1) socio-demographic and health characteristics; 2) drug-use practices and 3) willingness to receive IV buprenorphine treatment. The latter section included our two primary outcomes assessed using two questions: (1) *How would you rate your willingness to receive IV buprenorphine on a scale from 0 to 10?* (2) *How would you rate your willingness to receive IV buprenorphine on a scale from 0 to 10 if you had to come to a specialized addiction center to get it?*

### Statistical analyses

To identify factors associated with willingness to receive IV buprenorphine treatment, we performed an ordinal logistic regression model using the willingness score (0–10) as the dependent variable. Independent variables selection procedure was based on the following steps. First, we selected from our data a set of candidate variables based on literature review and experience of researchers and field workers. Second, we ran univariate analyses to estimate the association between our dependent variable and each of these pre-selected independent variables. In the univariate analyses, we used a *p* value threshold of *p* < 0.20 to identify variables eligible to enter into the multivariate model. This threshold was chosen to prevent the exclusion of potentially important explanatory variables. For the multivariate model variable selection, we used a backward elimination procedure to identify the explanatory variables, by keeping only those variables that significantly improved the model in terms of the likelihood ratio test and with a *p* value < 0.05. Finally, we used the likelihood-ratio test to ascertain whether proportionality of odds assumption was not violated.

### Qualitative material and dissemination booklet

At the end of the primary analyses of the PrebupIV survey [22], community-based workshops incorporating various participation stimuli were ran. These workshops took place in 6 participating centers and were moderated by community workers. Artworks (*n* = 22) and testimonies (*n* = 47) were collected from participants regarding their experiences with buprenorphine injection and drug use in general. For the present study, in order to illustrate and better understand our research findings from the quantitative analysis, we selected artworks and testimonies which specifically regarded buprenorphine injection and interest in IV treatment. All materials regarding these themes were screened by the study investigators and relevant ones were included in our analysis. This material collected during the workshops was not collected using classical qualitative methods and then, it could not be analyzed in-depth. However, this material is illustrative of our quantitative results and of people lived experience. This qualitative material was also used to build a dissemination booklet to share the study’s findings with its stakeholders. The booklet included the artworks and testimonies collected during the workshops and simplified scientific articles. It is available in French, both in paper and digital format.

## Results

### From the quantitative questionnaires

Among the 209 participants who reported regular buprenorphine injection, 21% were female and median (interquartile range (IQR)) age was 34 (28–41) years. Less than a third reported being employed and 39% reported having unstable housing. With regard to polydrug use, 76% reported the use of at least one non-opioid drug, 41% alcohol and 27% benzodiazepine. Sublingual buprenorphine was prescribed by a physician in 93% of cases. Median (IQR) daily buprenorphine doses was 11 mg (8–16), and median (IQR) number of daily injections was 3 (2–4). The main reported reasons for injecting were to avoid withdrawal or to feel good enough for daily functioning (59%), the pleasure of the act itself (23%) and to get high (15%). Eighty-four percent of participants reported experiencing more than 5 injection-related complications during their life, the most frequent being puffy hand syndrome (68%), thrombosis (59%) and abscesses (54%). Finally, one third of the sample reported a history of hepatitis C virus (HCV) infection. The mean score (SD) for general (i.e., restricted or not) willingness to receive IV buprenorphine was 8.0 (2.8), and 2.5 (2.9) for restricted dispensing in a specialized addiction center.

Table 1 shows the univariate and multivariate analyses of data on general willingness to receive IV buprenorphine. Univariate analyses showed several eligible variables for the multivariate analysis (i.e., *p* < 0.20): the pleasure of the act itself as the main reason for injecting, more than 5 lifetime injection-related complications, daily use of buprenorphine (i.e., dose and frequency), and injection of non-prescribed buprenorphine. Multivariate analysis showed that participants who reported more than 5 lifetime injection-related complications, those who used greater doses of buprenorphine and those who did not receive prescribed buprenorphine, were all more likely to be willing to receive buprenorphine.

**Table 1:** Factors associated with general willingness to receive intravenous buprenorphine treatment in the study sample

	*N* (%) or Median [IQR]	Univariate analysis *n* = 209			Multivariate analysis *n* = 197		
		OR	[95% CI]	*p*	AOR	[95% CI]	*p*
**Questionnaire**							
Online	162 (78)	0					
Face-to-face	47 (22)	0.97	[0.53; 1.77]	0.924			
**Gender**							
Male	164 (79)	0					
Female	43 (21)	0.75	[0.41; 1.35]	0.335			
**Age**							
For 1 year	34 [28–41]	0.99	[0.96; 1.02]	0.619			
**Stable housing**							
No	81 (39)	0					
Yes	127 (61)	0.72	[0.43; 1.22]	0.228			
**Employment**							
No	147 (70)	0					
Yes	62 (30)	0.76	[0.44; 1.30]	0.319			
**Duration of opioid use**							
For 1 year	8 [4–11]	1.01	[0.96; 1.06]	0.614			
**Duration of buprenorphine use**							
For 1 year	6 [4–10]	1.02	[0.97; 1.07.]	0.528			
**Daily buprenorphine dose**							
For 1 mg	11 [8–16]	1.04	[1.00;				
1.09]	*0.052*	1.05	[1.00; 1.10]	0.043			
**Daily injection frequency**							
For 1 injection	3 [2–4]	1.12	[0.98; 1.27]	0.086			
**Buprenorphine non-prescribed**							
No	187 (93)	0					
Yes	13 (7)	3.98	[1.09; 14.47]	0.036	4.82	[1.30; 17.85]	0.019
**Main reason for injecting buprenorphine**							
To get “high”	27 (15)	0					
To avoid withdrawal symptoms or to feel good enough for daily functioning	103 (59)	0.96	[0.44; 2.10]	0.929			
For the pleasure of the act	41 (23)	2.23	[1.08; 4.61]	0.030			
**Other non-opioid drugs used**							
No	49 (24)	0					
Yes	156 (76)	1.03	[0.56; 1.89]	0.934			
**Alcohol use**							
No	124 (59)	0					
Yes	85 (41)	0.99	[0.60; 1.67]	0.997			
**Lifetime number of injection-related complications (0–10)**							
≤ 5 complications	175 (84)	0					
> 5 complications	34 (16)	2.29	[1.08; 4.88]	0.031	2.28	[1.05; 4.93]	0.037
**Lifetime history of overdose**							
No	168 (80)	0					
Yes	41 (20)	1.26	[0.65; 2.47]	0.493			
**Self-reported HCV status**							
No	129 (66)	0					
Yes	66 (34)	0.87	[0.50; 1.53]	0.647			

Univariate and multivariate ordinal logistic regression models

*CI* confidence interval, *IQR* interquartile range, *AOR* adjusted odds ratio

In terms of willingness to receive IV buprenorphine restricted to delivery in a specialized addiction center, the only associated variable was non-prescribed buprenorphine injection (OR 4.05, 95% CI 1.17–14.03, *p* = 0.028).

### Qualitative material

**Fig. 1:**
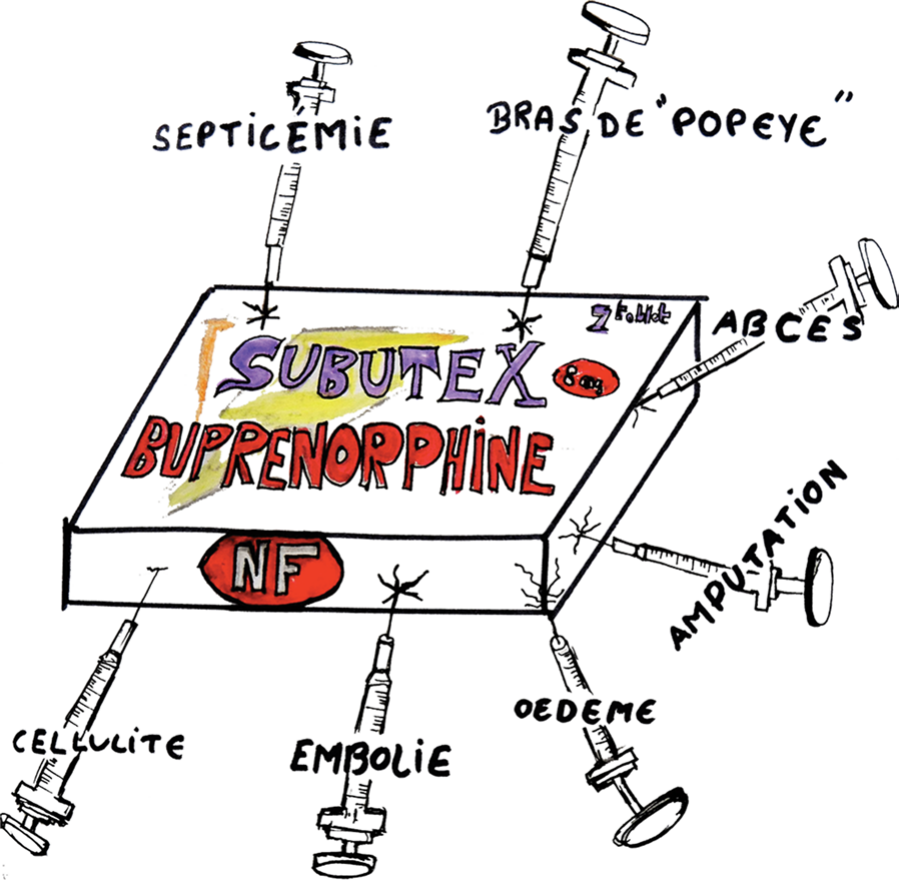
Artwork representing a buprenorphine box with harms related to its injection. Translation: septicaemia, “popeye” arm, abscesses, amputation, edema, embolism, cellulitis

**Fig. 2:**
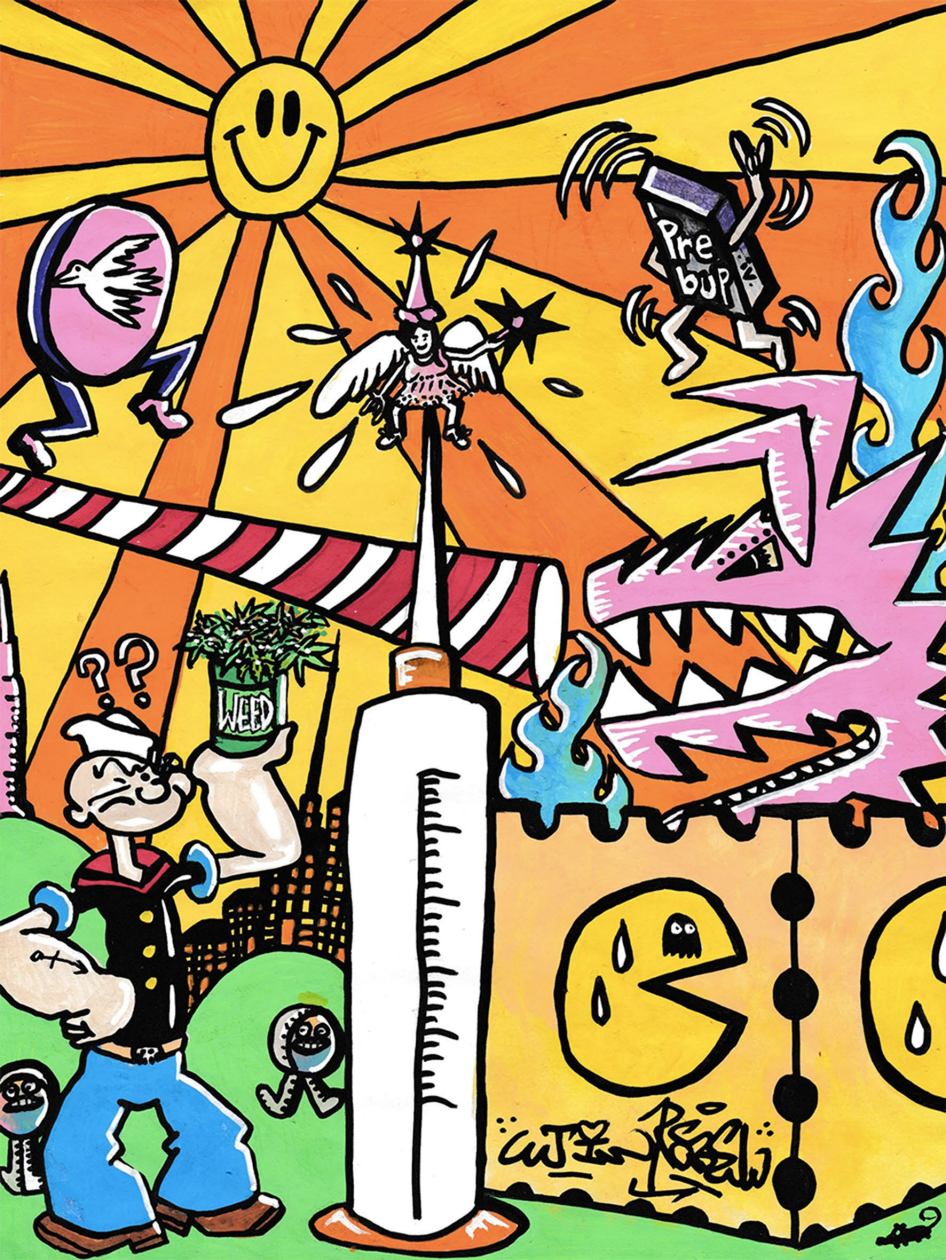
General artwork of the study. Popeye imagery refers to the common complication of buprenorphine injection characterized by persistent swelling of hands and forearms

**Fig. 3:**
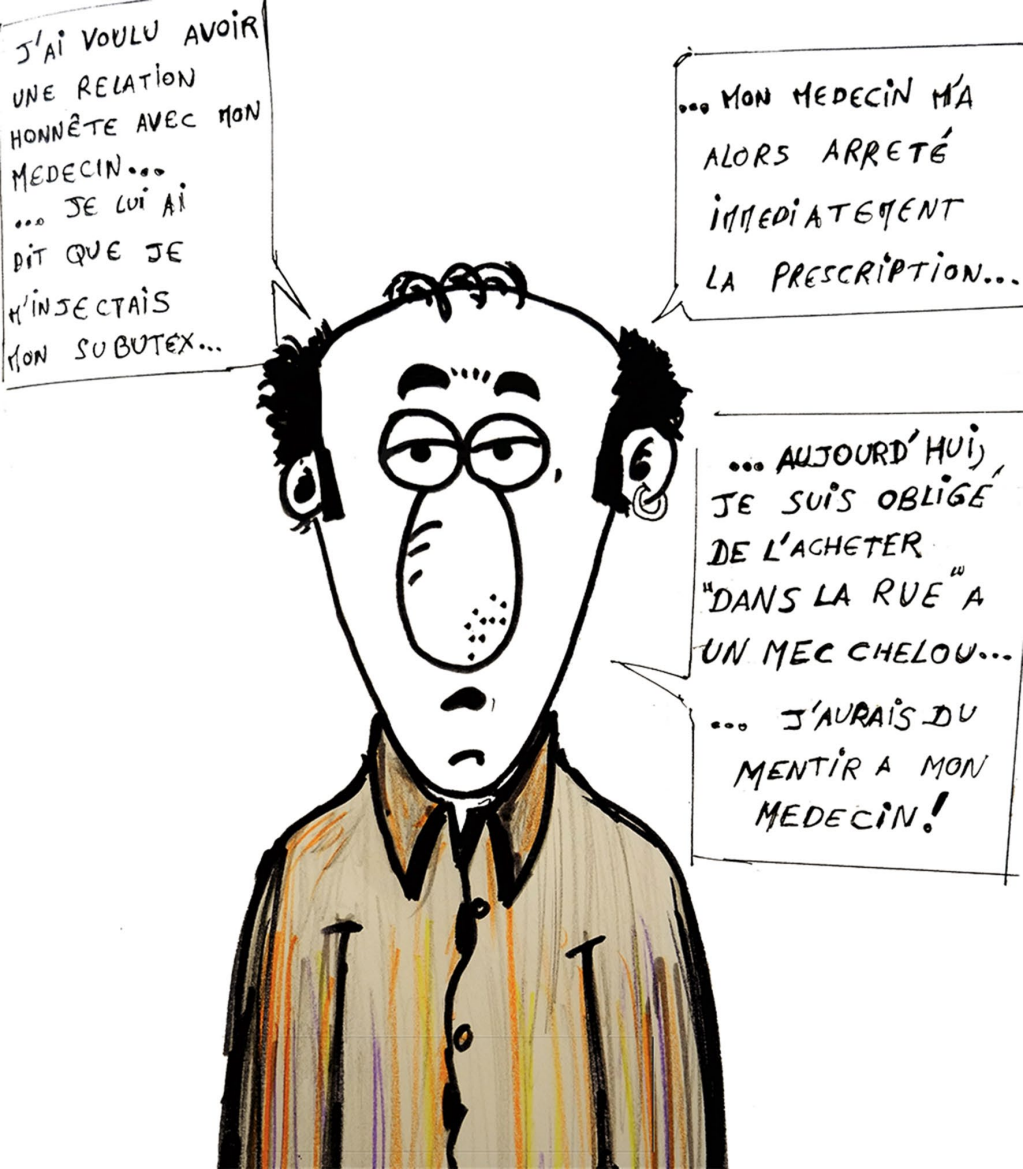
Artwork illustrating issues between physicians and users. Translation: “I wanted an honest relationship with my physician… I told him I was injecting my Subutex… My physician then stopped my prescription immediately… Today, I’m forced to buy it ‘in the street’ from a dodgy guy… I should have lied to my physician!

The users’ testimonies and artworks collected in workshops are useful to present in association with the quantitative results for illustrative purposes. Artworks described motivations to receive IV buprenorphine by depicting the complications associated with injecting buprenorphine tablets (Fig. 1), the “Popeye syndrome” (Fig. 2), and difficulties with physicians (Fig. 3). Users also described their experiences with buprenorphine injection with short testimonies. The most illustrative of these are presented below:

*“During dispensing in the prison’s healthcare unit, nurses caught me hiding my Subutex (because I wanted to inject it). To punish me, nurses reduced my dose by half.”; “The physician doesn’t want to prescribe Subutex to me if I inject it… So I’m lying to him.”; “When I inject Subutex, I’m seen as a junkie because I’m diverting the administration route.”; “I almost lost my hand, my leg and my arm because of abscesses caused by Subutex injections.”; “Ten years after having stopped injecting Subutex, my hands are still swollen (like “boxing gloves”).”*

## Discussion

This analysis showed that, among people with OUD who regularly inject buprenorphine sublingual tablets, the more willing individuals to receive IV buprenorphine treatment are those who experienced more detrimental outcomes with sublingual buprenorphine, namely lack of access to the medicine, insufficient dosage and injecting-related harms. This result is important to help determine which patients will be more suitable for IV buprenorphine treatment in future clinical trials. These findings show the importance of providing adequate treatment in terms of mode of administration (including intravenous one) and type of molecule (including buprenorphine). Previous researches have already demonstrated effectiveness of other injectable OAT with diacetylmorphine and hydromorphone and these are also implemented in other countries [19, 20]. Our results also showed that participants were less willing to receive buprenorphine IV treatment if restricted to supervision in specialized centers. This result suggests the need for treatment modalities which are not overly restrictive for people with OUD. Under certain circumstances and after rigorous clinical evaluation, more flexible options might be offered to patients given the safety profile of buprenorphine. Low-threshold services intended to overcome treatment accessibility or design barriers (e.g., admission criteria, duration of treatment) have been successfully implemented for oral-based OAT and need to be considered for injectable OAT [23, 24].

Willingness to receive prescribed IV buprenorphine treatment differed according to different factors. First, people who used non-prescribed buprenorphine were more willing than those who were prescribed it. This correlate was also identified for willingness for supervised treatment. This suggests that an IV formulation could be more attractive to individuals not being treated for their OUD. Since it has already been demonstrated that medical follow-up is crucial to help individuals with OUD obtain access to stable treatment, adequate prevention and global care, this finding shows the potential impact of IV buprenorphine treatment in attracting buprenorphine injectors into healthcare [25]. Testimonies from participants provided more information about this result, as some reported that their prescription was stopped after disclosing to their physician that they injected their buprenorphine. Stigma towards people who inject drugs (PWID) is frequent in healthcare settings and is associated with negative health outcomes and limited access to OAT [26]. Implementing prescribed IV buprenorphine as a new treatment strategy might reduce stigma associated with buprenorphine misuse and facilitate access to care for highly stigmatized individuals.

Second, participants who reported more injection-related complications were more interested in IV buprenorphine treatment. Since the two most frequent complications experienced by the participants (i.e., puffy hand syndrome and thrombosis) are closely related with injection of sublingual buprenorphine, this suggests that participants made the connection between this practice and the complications they experienced. This aspect was also particularly present in artworks and testimonies from the workshops. Users often linked injection of sublingual buprenorphine to specific complications, in particular with puffy hand syndrome, which was drawn using the metaphor and imagery of Popeye. These harms could be significantly reduced if a specific IV formulation were to become authorized. Nevertheless, appropriate education about safe injection practices would also need to be implemented to prevent inherent injection-related risks, such as HIV and HCV transmission or local infections. Various programs, such as AERLI in France, have already shown the effectiveness of educational interventions in reducing unsafe injecting practices, and could be implemented as therapeutic patient education in this context [27, 28]. In addition, as individual-based interventions may have limited impact, intervention targeting environmental risk factors (e.g., social deprivation, criminalization, stigma) should be encouraged to fully address people health issues [29].

With regard to the dosis of buprenorphine used, in our sample, median daily use was 11 mg, which is roughly equivalent to 35 mg of sublingual buprenorphine, as the bioavailability of sublingual buprenorphine is approximately 30%, as opposed to an estimated 100% for IV administration [30]. In France, the maximum daily dose authorized for OUD management is 24 mg, suggesting that one motivation for people to inject buprenorphine is their need for higher doses. This result is consistent with previous studies showing the link between buprenorphine injection and inadequate patient-perceived clinical dosing [31]. Our findings also showed that participants who used higher buprenorphine doses were more willing to receive IV buprenorphine. This result indicates that future clinical trials evaluating IV buprenorphine treatment should include high doses to meet the needs of regular buprenorphine injectors. In a previous study, Umbricht et al*.* compared the pharmacodynamics of up to 16 mg of both sublingual and IV buprenorphine among 6 long-term opioid users. They showed that buprenorphine exhibits a ceiling effect for both subjective and cardiorespiratory measures, indicating good safety of IV buprenorphine even at high doses [32]. Nonetheless, their results should be interpreted with caution due to small sample size, and more research is needed to assess safety of IV buprenorphine.

The main patient-declared reasons for injecting buprenorphine were therapeutic and not recreational in nature. This result is in line with previous studies showing that the main reasons cited for injecting buprenorphine were to treat dependence and the desire to avoid withdrawal effects [13, 14]. Nevertheless, almost a quarter of our sample reported the pleasure of the act as the main reason for injecting, suggesting a strong culture of injection among PWID. This could be related to the concept of “needle fixation” which suggests that the injecting process is a part of the subjective drug effects (e.g., pleasure, relief, etc.) [33]. Injectable OAT with diacetylmorphine, hydromorphone, and potentially buprenorphine, are therefore all well-suited for people unable to stop injecting.

Finally, the art-based workshops carried out after completing the quantitative questionnaires were useful to collect users’ experiences and views. The booklet subsequently created was also useful to disseminate our research findings. Researchers have shown that art-based workshops are very well-suited to engage people to speak about sensitive topics [34]. However, the material collected in the workshops was informal and cannot be used as proper qualitative data, but only to illustrate our results.

### Limitations

Some study limitations have to be acknowledged. First, our study was conducted before European approval for prolonged-release formulations in 2018. Accordingly, if it were to be repeated today, willingness for IV buprenorphine treatment might be lower as people would have heard about these formulations. Having said that, these treatments seem more suitable for stable patients than treatment-refractory and entrenched injectors who constituted our study population. Moreover, in 2020, these treatments are not yet available in France and concerns arose about limited data regarding their clinical efficacy and adequacy with the French model of OAT [35]. For these reasons, we are confident in the relevance of our data. Second, our study relied on self-reported data which is potentially subject to social desirability bias. However, the reliability of self-reports in the drug-using population has already been documented [36].

## Conclusion

This study showed that willingness for IV buprenorphine treatment was very high among people who regularly injected sublingual buprenorphine (whether prescribed or not). Those who used non-prescription buprenorphine, those who reported higher doses of buprenorphine use, and those who had experienced more injecting-related complications, were all more willing to receive prescribed IV buprenorphine treatment. Both these results are clear not only from the quantitative questionnaires but also from the informal qualitative material collected (*i.e.*, from artwork and testimonies during the workshops). Our analysis highlights the importance of needs-based individualized treatment options, and our data provide indispensable information for the next step of this project, which is to implement a clinical evaluation of IV buprenorphine treatment. Furthermore, future research will need to evaluate whether access to medicalized IV OAT has the potential to reduce social, structural and self-stigma associated with injecting drug use.

## Data Availability

The datasets used and/or analyzed during the current study are available from the corresponding author on reasonable request.

## Abbreviations

HCV: Hepatitis C virus
HIV: Human immunodeficiency virus
IV: Intravenous
OAT: Opioid agonist treatment
OUD: Opioid use disorders
PWID: People who inject drugs
