# Correction to: Individual and structural correlates of willingness for intravenous buprenorphine treatment among people who inject sublingual buprenorphine in France

**DOI:** 10.1186/s12954-021-00465-9

**Published:** 2021-02-19

**Authors:** Salim Mezaache, Patrizia Carrieri, Laelia Briand‑Madrid, Virginie Laporte, Alain Morel, Daniela Rojas Castro, Perrine Roux

**Affiliations:** 1 INSERM, IRD, SESSTIM, Sciences Economiques & Sociales de La Sante & Traitement de L’information Medicale, Aix-Marseille Univ, Marseille, France; 2 ORS PACA, Observatoire Regional de La Sante Provence-Alpes-Cote D’Azur, Marseille, France; 3 Aides, Pantin, France; 4 Association Oppelia, Paris, France; 5 Laboratoire de Recherche Communautaire Coalition PLUS, Pantin, France

## Correction to: Mezaache et al. Harm Reduct J (2021) 18:11 https://doi.org/10.1186/s12954-021-00460-0

Following publication of the original article [1], the authors identified an error in Table 1.

The correct table is given below.

**Table 1:** Factors associated with general willingness to receive intravenous buprenorphine treatment in the study sample

	N (%) or Median [IQR]	Univariate analysis n = 209			Multivariate analysis n = 197		
		OR	[95CI%]	*p*	AOR	[95CI%]	*p*
Questionnaire							
Online	162 (78)	0					
Face-to-face	47 (22)	0.97	[0.53; 1.77]	*0.924*			
Gender							
Male	164 (79)	0					
Female	43 (21)	0.75	[0.41; 1.35]	*0.335*			
Age							
For 1 year	34 [28–41]	0.99	[0.96; 1.02]	*0.619*			
Stable housing							
No	81 (39)	0					
Yes	127 (61)	0.72	[0.43; 1.22]	*0.228*			
Employment							
No	147 (70)	0					
Yes	62 (30)	0.76	[0.44; 1.30]	*0.319*			
Duration of opioid use							
For 1 year	8 [4–11]	1.01	[0.96; 1.06]	*0.614*			
Duration of buprenorphine use							
For 1 year	6 [4–10]	1.02	[0.97; 1.07.]	*0.528*			
Daily buprenorphine dose							
For 1 mg	11 [8–16]	1.04	[1.00; 1.09]	*0.052*	1.05	[1.00; 1.10]	*0.043*
Daily injection frequency							
For 1 injection	3 [2–4]	1.12	[0.98; 1.27]	*0.086*			
Buprenorphine non-prescribed							
No	187 (93)	0					
Yes	13 (7)	3.98	[1.09; 14.47]	*0.036*	4.82	[1.30; 17.85]	*0.019*
Main reason for injecting buprenorphine							
To get “high”	27 (15)	0					
To avoid withdrawal symptoms or to feel good enough for daily functioning	103 (59)	0.96	[0.44; 2.10]	*0.929*			
For the pleasure of the act	41 (23)	2.23	[1.08; 4.61]	*0.030*			
Other non-opioid drugs used							
No	49 (24)	0					
Yes	156 (76)	1.03	[0.56; 1.89]	*0.934*			
Alcohol consumption							
No	124 (59)	0					
Yes	85 (41)	0.99	[0.60; 1.67]	*0.997*			
Lifetime number of injection-related complications (0–10)							
≤ 5 complications	175 (84)	0					
> 5 complications	34 (16)	2.29	[1.08; 4.88]	*0.031*	2.28	[1.05; 4.93]	*0.037*
Lifetime history of overdose							
No	168 (80)	0					
Yes	41 (20)	1.26	[0.65; 2.47]	*0.493*			
Self-reported HCV status							
No	129 (66)	0					
Yes	66 (34)	0.87	[0.50; 1.53]	*0.647*			

The original article has been corrected.
